# Poor Walking Speed Is Associated With Higher Segment-Specific Arterial Stiffness in Older Adult Japanese Community Dwellers: A Cross-Sectional Study

**DOI:** 10.3389/fphys.2020.587215

**Published:** 2020-11-23

**Authors:** Noriko Ogawa, Chika Nanayama Tanaka, Minenori Ishido, Tomohiro Nakamura, Masato Nishiwaki

**Affiliations:** ^1^Graduate Course in Applied Chemistry, Environmental and Biomedical Engineering, Osaka Institute of Technology, Osaka, Japan; ^2^Faculty of Nursing, Setsunan University, Osaka, Japan; ^3^Faculty of Engineering, Osaka Institute of Technology, Osaka, Japan

**Keywords:** arteriosclerosis, gait ability, cardiovascular disease, cardio-ankle vascular index, pulse wave velocity

## Abstract

Walking speed as one index of gait ability is an important component of physical fitness among older adults. Walking speed-arterial stiffness relationships have been studied, but whether poor walking speed is associated with higher segment-specific arterial stiffness in older adults is unclear. We thus aimed to examine the relationship between walking speed and segmental arterial stiffness among older community dwellers. This study was a cross-sectional study of 492 older Japanese community dwellers (age range, 65 to 96 years). Heart-brachial PWV (hbPWV), brachial-ankle PWV (baPWV), heart-ankle PWV (haPWV), and cardio-ankle vascular index (CAVI) were used as arterial stiffness indices. Walking speed, strength, flexibility, and cognitive function were also assessed. The participants were categorized into low (Slow), middle (Middle), and high (Fast) tertiles according to walking speed. The CAVI and baPWV were significantly lower in Fast than in Slow. Significant decreasing trends in CAVI and baPWV and a tendency toward decreasing trend in haPWV were observed from Slow to Fast, whereas hbPWV did not significantly differ among tertiles and no trend was evident. The results remained significant after normalizing CAVI and PWVs for multicollinearity of arterial stiffness indices and major confounding factors, such as age, gender, body mass index, blood pressure, cognitive function, and each physical fitness. Therefore, these findings suggest that poor walking speed is associated with higher segment-specific arterial stiffness of the central and lower limbs, but not of upper, in older adult community dwellers.

## Introduction

Pulse wave velocity (PWV) and cardio-ankle vascular index (CAVI) are widely used as clinical indicators of arterial stiffness (Tanaka and Safar, 2005; Shirai et al., 2011). Increased arterial stiffness has been identified as an independent risk factor for future cardiovascular diseases or mortality (Laurent and Boutouyrie, 2007; Vlachopoulos et al., 2010; Vlachopoulos et al., 2012; Ohkuma et al., 2017). Arterial stiffness progressively increases with advancing age even among healthy individuals (Tomiyama et al., 2003; Nishiwaki et al., 2014b, 2019b), but the degree is modulated by levels of physical fitness or physical activity (PA). In fact, higher levels of physical fitness or PA are associated with less arterial stiffness among older people (Yamamoto et al., 2009; Gando et al., 2010; Nishiwaki et al., 2014b; Gando et al., 2016), and regular PA can also reduce arterial stiffness even among older people (Miura et al., 2015; Nishiwaki et al., 2018). Maintaining physical fitness and habitually participating in PA are thus of paramount importance for preventing and improving arterial stiffness among older adults.

Walking speed as one index of gait ability is an important component of physical fitness, especially among older adults (Barak et al., 2006), is generally considered to predict future cardiovascular diseases, disability, or mortality (Al Snih et al., 2002; Cesari et al., 2005, 2009; Rolland et al., 2006; Ostir et al., 2007; Mozaffarian et al., 2008; Dumurgier et al., 2009; Sattelmair et al., 2010), and is recognized as important for preventing frailty (Fried et al., 2001; Shimada et al., 2015). Although cardiorespiratory fitness, strength, and flexibility are widely known to be associated with arterial stiffness (Vaitkevicius et al., 1993; Yamamoto et al., 2009; Nishiwaki et al., 2014b; Tanisawa et al., 2015; Gando et al., 2016), some studies have examined relationships between gait ability and arterial stiffness in older adults (Brunner et al., 2011; Watson et al., 2011; Gonzales, 2013). The Whitehall II Study identified an inverse correlation between walking speed and carotid-femoral PWV (cfPWV) (Brunner et al., 2011). The study of Gonzales also reported that lower gait performance is significantly associated with higher cfPWV (Gonzales, 2013). Therefore, these findings indicate that walking speed is related to aortic arterial stiffness (cfPWV) in older adults and understanding such relationship could help partially to explain why gait ability can predict cardiovascular diseases and mortality.

Although aortic arterial stiffness has been generally proposed as the reference standard, brachial-ankle PWV (baPWV), heart-brachial PWV (hbPWV), and CAVI are attracting increasing attention (Kim et al., 2019; Sugawara et al., 2019). These parameters do not require higher special technique (*i.e.*, placement of transducers) and are significantly associated with aortic arterial stiffness (Kim et al., 2019; Sugawara et al., 2019). However, PWV-assessed arterial stiffness would differ in regards to measurement segmentation (*i.e*., central vs. peripheral and active limb vs. non-active limb) (Kume et al., 2020; Nishiwaki et al., 2020). Indeed, Avolio et al. (1985) and Tomiyama et al. (2003) indicate that age-related increase in arterial stiffness can be greater in aortic and leg than in arm. Recent studies also identified that single-exercise (cycling, resistance exercise, and stretching) reduces arterial stiffness only in the exercised leg, but not in the control non-exercised leg (Sugawara et al., 2004; Heffernan et al., 2006; Yamato et al., 2017). Interestingly, a previous study indicates that slower gait speed was associated with higher PWV in older adults with peripheral artery disease (PAD), but not in older adults without PAD (Watson et al., 2011). Thus, these findings raise the possibility that walking speed relates to statuses of artery, especially in central and lower limbs or primarily exercise-related limbs in older adults. Therefore, further detailed studies are required to examine the relationship between walking speed and segmental arterial stiffness. However, these points have not been addressed in community dwelling older adults as far as we can ascertain.

With this information as background, we hypothesized that walking slowly reflects site-specific higher arterial stiffness among older adults. Therefore, this cross-sectional study aimed to examine the relationship between walking speed and segmental arterial stiffness among older community dwellers in Japan.

## Methods

### Participants

Japanese community dwellers aged ≥ 65 years in Osaka and Kawakami village in Nara Prefecture were recruited using local advertisements and referrals from public offices. The data collection period was from September 2014 to December 2019, total 583 participants were recruited in this cross-sectional study. We excluded 91 participants due to missing data associated with arterial stiffness and physical fitness (*n* = 14, 2.4%), refusal to cooperate (*n* = 5, 0.9%), pain (*n* = 4, 0.6%), dementia (score of the Cognitive Assessment for Dementia, iPad version 2 [CADi2] ≤ 5) (*n* = 28, 4.8%), chronic diseases that affected walking (*n* = 2, 0.3%), features of peripheral arterial disease (ankle-brachial index [ABI] < 0.90) (*n* = 20, 3.4%), and technical errors associated with measuring arterial stiffness such as an undetectable pulse wave (*n* = 18, 3.1%). Data from 492 (male, *n* = 166; female, *n* = 326; age, 65–96 years) older Japanese community dwellers who met the inclusion criteria were analyzed. In accordance with previously reported methods (Dumurgier et al., 2009), the participants were categorized according to low (≤ 1.54 m/s in men and ≤ 1.49 m/s in women; Slow), middle (1.55 – 1.83 m/s in men and 1.50 – 1.78 m/s in women; Middle), or high (≥ 1.84 m/s in men and ≥ 1.79 m/s in women; Fast) tertiles based on each walking speed. The purpose, procedures, and risks of the study were explained to the recruits, all of whom provided written informed consent to participate. The Human Ethics Committee at the Osaka Institute of Technology reviewed and approved the study (2016-7 and 2018-1), which was conducted according to the guidelines of the Declaration of Helsinki.

### Measurements

The participants abstained from vigorous exercise for at least 24 h before, cigarette smoking and medications on the day, and caffeine and food for 4 h before testing. We measured the height and weight of participants while wearing light clothing, then body mass index (BMI) was calculated as weight divided by height squared (Nishiwaki et al., 2014a). Waist circumference (WC) was measured around the abdomen at the level of the navel at the late expiratory phase using a tape measure (Nishiwaki et al., 2017b). We also determined smoking status, use of anti-hypertensive or anti-hyperlipidemic medications, and exercise status in face-to-face interviews (as yes or no answers). All parameters were measured in a quiet air-conditioned room at 22°C – 24°C.

### Arterial Stiffness, Blood Pressure, and Heart Rate

After resting for 15 min, segmental PWV, blood pressure (BP), and heart rate (HR) were measured in the supine position using a VS-1500AE/AN semi-automated device (Fukuda Denshi, Japan) (Nishiwaki et al., 2017a, c, 2019a). Electrocardiography (ECG), heart sounds, PWV, and BP were assessed in the supine position. Electrodes for ECG were placed on both wrists, and a microphone was placed at the sternum for phonocardiography. HR was automatically calculated from the R-R intervals on ECG. Cuffs were wrapped around both brachial upper arms and ankles and connected to a volume-plethysmographic sensor that determines volume pulse form and an oscillometric pressure sensor that measures BP. Ankle-brachial index (ABI) was obtained by dividing ankle SBP by brachial SBP. Cardio-ankle vascular index (CAVI) was automatically calculated from 5 pulse-wave signals using the following formula: CAVI = a [(2ρ/PP) × ln (SBP/DBP) × PWV^2^] + b, where DBP is diastolic blood pressure, PP is SBP - DBP, ρ is the blood density, and a and b are constants (Shirai et al., 2011; Nishiwaki et al., 2014b). The heart-brachial PWV (hbPWV), brachial-ankle PWV (baPWV), and heart-ankle PWV (haPWV) were also calculated by dividing the distance between the recording sites by the transit times, as described (Yamashina et al., 2002; Nishiwaki et al., 2015). The means of the left and right brachial BP, PWV, and CAVI values in each participant were subsequently analyzed. The coefficients of variation (CV) that reflect the reproducibility of measurements, were 4.2 ± 0.6%, 2.7 ± 0.3%, 2.6 ± 0.6%, and 3.6 ± 0.6% for hbPWV, baPWV, haPWV, and CAVI, respectively (Nishiwaki et al., 2017a, c, 2019a).

### Cognitive Function and Physical Fitness Test

Cognitive function was assessed using the CADi2 as described (Onoda et al., 2013). The CADi2 consists of the following items: immediate recognition memory for three words, semantic memory, categorization of six objects, subtraction, backward repetition of digits, cube rotation, pyramid rotation, trail-making A, trail-making B, and delayed recognition memory for three words. Scores for the CADi2 significantly correlate with Mini-Mental State Examination (MMSE) scores that serve as the gold-standard of cognitive function assessment (Onoda et al., 2013). If MMSE cutoff of 23 points is applied, a cut point corresponding the score is 5 and serve as a useful tool for assessing dementia in Japanese populations (Onoda et al., 2013).

Handgrip strength was measured in duplicate using a dynamometer (T.K.K.5001 Grip-A; Takei Scientific Instruments Co., Ltd., Tokyo, Japan) and mean left and right values were analyzed (Nishiwaki et al., 2014b, 2015, 2017c). Knee extension strength was isometrically measured in duplicate using a dynamometer (TKK-5715; Takei Scientific Instruments Co. Ltd., Tokyo, Japan) and mean left and right values were analyzed. Trunk flexibility was measured with the sit-and-reach test using a T-283 device (Toei Light, Tokyo, Japan) as described (Nishiwaki et al., 2014b, 2015). The 6 m walk test proceeded as follows (Dumurgier et al., 2009; Nishiwaki et al., 2018, 2019b). The participants started to walk 3 m before a start line to exclude the duration of acceleration from normal walking speed, then walked as fast as possible without running for 6 m. The walk time during the 6 m walking was measured in duplicate using a stopwatch and the mean values were used. Walking speed was calculated as the ratio between distance and time. A previous large prospective cohort study has demonstrated that slow speed of the short distance maximum walking is associated with cardiovascular death in older adults (Dumurgier et al., 2009). Because the walk test of short distance (6 m) is simple to administer in a short time and convenient to measure in a room, the present study used the test as an indicator of gait speed. In order to compare the observed maximum walking speed with the theoretical optimal walking speed of the elderly (OWS), the locomotor rehabilitation index (LRI) was also calculated by using following formula; LRI (%) = walking speed (maximum) / $\sqrt{0.25\cdot 9.81\cdot \mathrm{participants}\,\text{’}\,\mathrm{height}\cdot 0.54}\,\cdot$ 100 (Gomenuka et al., 2019). All daily CVs were < 10% (Nishiwaki et al., 2014b, 2015, 2017c, 2018, 2019b).

### Physical Activity

In 338 (108 males and 229 females) of limited participants, physical activity (PA) data during one week were assessed by a single-axis activity monitor (Lifecorder PLUS; Suzuken Co., Ltd., Aichi, Japan) under sealed conditions (uninformed measured values), as previously described (Nishiwaki et al., 2017b, 2018). A valid day was defined as wearing the monitor for > 10 h (Troiano et al., 2008). The length of time that the monitor was worn was determined by subtracting the length of time during which it was not worn (non-wear time) from 24 h. Non-wear time was defined as an interval of at least 60 consecutive minutes of zero activity intensity counts (Troiano et al., 2008). Days when the equipment was not worn were excluded from analysis, and data from participants who had four or more valid days per week were subsequently analyzed. Based on the study of Kumahara et al. (2004), we obtained steps (steps/day) and the duration of daily PA corresponding to 1.5-2.9 METs (light), 3.0-5.9 METs (moderate), and > 6.0 METs (vigorous), and to < 1.4 METs by subtracting the sum of the duration of light, moderate and vigorous PA from 1440 min (inactive time).

### Statistics

Continuous data were analyzed using one-way ANOVA and MANCOVA. In particular, the analysis of MANCOVA with adjusted model 1 were normalized multicollinearity of arterial stiffness indices and confounding factors such as age, gender, BMI, mean BP (MBP), HR, cognitive function, strength, flexibility, smoking status, exercise status, anti-hypertensive medication, and anti-hyperlipidemic medication. The analysis of MANCOVA with adjusted model 2 were also normalized factors of model 1, moderate PA time, and vigorous PA time. Significant F values were assessed using post hoc tests with the Bonferroni correction to identify significant differences among mean values. Trends were analyzed using linearity tests and weighted P values were adjusted for sample size. Differences in non-parametric variables were analyzed using Kruskal–Wallis tests followed by Schefféì tests. The relationships between walking speed or LRI and each arterial stiffness indices were assessed using univariate regression analyses and forced entry multiple regression analyses. All data were statistically analyzed using SPSS 25.0J (IBM SPSS Japan, Japan). Data are presented as means ± SE. Differences and relationships were considered significant at *P* < 0.05.

## Results

Table 1 shows the characteristics of the participants divided into tertiles according to walking speed. Age, height, and weight significantly differed and trends were evident among the tertiles. Although systolic BP and MBP did not differ among the tertiles, diastolic BP and ABI were significantly higher and pulse pressure was significantly lower in Fast group than in Slow group. Cognitive function and sit-and-reach were significantly better in Middle and Fast groups than in Slow group. Walking speed, LRI, handgrip, knee extension strength, time spent of moderate PA, and steps were significantly higher in Middle and Fast groups than in Slow group, and notably higher in Fast group than in Middle group. A significant increasing trend was evident in cognitive function, physical fitness, steps, and daily time spent in each physical activity from Slow to Fast.

**TABLE 1:** Characteristics of the participants.

Parameters	All	Slow group	Middle group	Fast group	Difference P	Trend P
	*n* = 492	*n* = 162	*n* = 165	*n* = 165	–	–
Number of Women (%)	326 (66.3)	107 (66.0)	109 (66.1)	110 (66.7)	=0.991	–
Age, yrs	76.4 ± 0.3	79.5 ± 0.5	76.3 ± 0.5*	73.5 ± 0.4*^†^	< 0.001	< 0.001
Height, cm	153.8 ± 0.4	151.3 ± 0.7	154.0 ± 0.6*	156.0 ± 0.7*	< 0.001	< 0.001
Weight, kg	53.9 ± 0.4	52.7 ± 0.8	53.3 ± 0.8	55.7 ± 0.8*	= 0.018	= 0.007
Body mass index, kg/m^2^	22.7 ± 0.1	23.0 ± 0.3	22.4 ± 0.2	22.8 ± 0.2	= 0.245	= 0.558
Waist circumference, cm	83.3 ± 0.4	85.1 ± 0.8	82.0 ± 0.7*	83.0 ± 0.7	= 0.011	= 0.047
Systolic BP, mmHg	141 ± 1	142 ± 1	141 ± 1	141 ± 2	= 0.920	= 0.955
Diastolic BP, mmHg	83 ± 1	82 ± 1	82 ± 1	85 ± 1*^†^	= 0.004	= 0.003
Mean BP, mmHg	108 ± 1	108 ± 1	107 ± 1	109 ± 1	= 0.572	= 0.492
Pulse Pressure, mmHg	58 ± 1	60 ± 1	59 ± 1	56 ± 1*	= 0.040	= 0.013
Heart rate, beats/min	71 ± 1	70 ± 1	71 ± 1	71 ± 1	= 0.451	= 0.274
Ankle-brachial index, unit	1.13 ± 0.01	1.12 ± 0.01	1.13 ± 0.01	1.15 ± 0.01*	< 0.001	< 0.001
Walking speed, m/s	1.66 ± 0.02	1.30 ± 0.01	1.65 ± 0.01*	2.03 ± 0.02*^†^	< 0.001	< 0.001
Locomotor rehabilitation index, %	116.3 ± 1.1	92.0 ± 1.0	115.9 ± 0.5*	141.2 ± 1.2*^†^	< 0.001	< 0.001
Handgrip strength, kg	25.1 ± 0.4	22.5 ± 0.6	25.3 ± 0.6*	27.4 ± 0.6*^†^	< 0.001	< 0.001
Knee extension strength, kg	23.6 ± 0.4	19.2 ± 0.6	23.9 ± 0.6*	27.8 ± 0.7*^†^	< 0.001	< 0.001
Sit-and-reach, cm	30.3 ± 0.4	27.7 ± 0.8	30.8 ± 0.7*	32.4 ± 0.8*	< 0.001	< 0.001
Cognitive function, scores	8.6 ± 0.1	8.2 ± 0.1	8.7 ± 0.1*	9.0 ± 0.1*	< 0.001	< 0.001
Number of smokers (%)	29 (5.9)	11 (6.8)	12 (7.3)	6 (3.6)	= 0.296	–
Number of participants with exercise habits (%)	284 (57.7)	83 (51.2)	96 (58.2)	105 (63.6)	= 0.074	–
Number of participants using HTM (%)	195 (39.6)	70 (43.2)	70 (42.4)	55 (33.3)	= 0.107	–
Number of Participants using HLM (%)	148 (30.1)	48 (29.6)	51 (30.9)	49 (29.7)	= 0.941	–
Daily time spent in physical activity	*n* = 337	*n* = 108	*n* = 107	*n* = 122	–	–
Light, min/day	680 ± 6	653 ± 12	682 ± 10	702 ± 10*	= 0.007	= 0.002
Moderate, min/day	14 ± 1	8 ± 1	14 ± 1*	18 ± 1*^†^	< 0.001	< 0.001
Vigorous, min/day	0.8 ± 0.2	0.3 ± 0.1	1.0 ± 0.5	1.0 ± 0.1	= 0.151	= 0.105
Inactivity, min/day	746 ± 7	778 ± 13	744 ± 11	719 ± 10*	= 0.001	< 0.001
Steps, steps/day	5850 ± 169	4431 ± 285	5834 ± 280*	7121 ± 261*^†^	< 0.001	< 0.001

*BP, blood pressure; HTM, anti-hypertensive medication; HLM, anti-hyperlipidemic medication. Date are mean ± SE. ^∗^P < 0.05 vs. Slow group, ^†^P < 0.05 vs. Middle group.*

**FIGURE 1:**
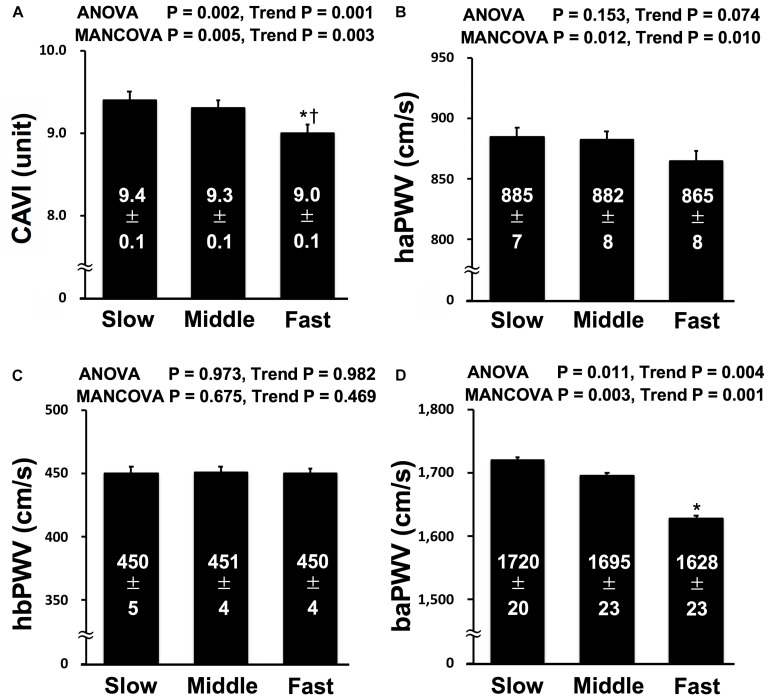
Comparisons of CAVI **(A)**, haPWV **(B)**, hbPWV **(C)**, and baPWV **(D)** in each tertile. CAVI, cardio-ankle vascular index; haPWV, heart-ankle pulse wave velocity; hbPWV, heart-brachial pulse wave velocity; baPWV, brachial-ankle pulse wave velocity; Slow, lowest tertile (≤ 1.54 m/s in men and ≤ 1.49 m/s in women); Middle, middle tertile (1.55 m/s to 1.83 m/s in men and 1.50 m/s to 1.78 m/s in women); Fast, highest tertile (≥ 1.84 m/s in men and ≥ 1.79 m/s in women). MANCOVA; the model was adjusted multicollinearity of arterial stiffness indices and confounding factors such as age, gender, body mass index, mean blood pressure, heart rate, cognitive function, handgrip strength, flexibility, smoking status, exercise status, anti-hypertensive medication, and anti-hyperlipidemic medication. *P < 0.05 vs. Slow; ^†^*P* < 0.05 vs. Middle.

Figure 1 shows the results for CAVI and PWVs in each group. The findings of ANOVA showed significantly lower CAVI and baPWV in Fast group than in Slow group, and the CAVI of Fast group was also lower than that in Middle group. Significant decreasing trends in baPWV and CAVI and a tendency toward decreasing trend in haPWV were observed from Slow to Fast, whereas hbPWV did not significantly differ among groups or exhibit any trends. MANCOVA of model 1 also indicates that the results of ANOVA did not change after normalizing CAVI, baPWV, and hbPWV but significantly changed after adjusting haPWV, which was lower in Fast group than in Slow and Middle groups, for multicollinearity of arterial stiffness indices and confounding factors such as age, gender, BMI, MBP, HR, cognitive function, handgrip strength, flexibility, smoking status, exercise status, anti-hypertensive medication, and anti-hyperlipidemic medication. For the limited participants, the analysis of MANCOVA of model 2 indicates that the results remained significant after normalizing CAVI and PWVs for moderate PA time and vigorous PA time in addition to factors of model 1.

The results of the univariate regression analysis indicate that walking speed correlated especially with handgrip strength (*r* = 0.365, *P* < 0.001), knee extension strength (*r* = 0.485, *P* < 0.001), flexibility (*r* = 0.183, *P* < 0.001), cognitive function (*r* = 0.240, *P* < 0.001), CAVI (*r* = −0.143, *P* = 0.001) and baPWV (*r* = −0.143, *P* = 0.002), but not with haPWV (*r* = −0.069, *P* = 0.128) and hbPWV (*r* = 0.043, *P* = 0.337). In order to determine the physical factors predicting walking speed in older adults, we further performed multiple regression analysis. In addition to BMI, knee extension strength, flexibility, and cognitive function, the analyses identified CAVI and baPWV were significant independent factors that regulate walking speed and haPWV tended to be an independent factor, but hbPWV were not (Table 2). LRI also correlated with CAVI (*r* = −0.165, *P* < 0.001) and baPWV (*r* = −0.138, *P* = 0.002). However, LRI did not correlate with haPWV (*r* = −0.088, *P* = 0.050) and hbPWV (*r* = 0.004, *P* = 0.936).

**TABLE 2:** Multiple-regression analyses of each arterial stiffness index and physical factors affecting walking speed.

		β	*P*-value
CAVI (*r* = 0.545, adjusted *r*^2^ = 0.288, *P* < 0.001)			
	Body mass index	−0.124	= 0.003
	Strength	0.470	< 0.001
	Flexibility	0.150	< 0.001
	Cognitive function	0.119	= 0.005
	CAVI	−0.111	= 0.008
haPWV (*r* = 0.539, adjusted *r*^2^ = 0.283, *P* < 0.001)			
	Body mass index	−0.117	= 0.006
	Strength	0.476	< 0.001
	Flexibility	0.154	< 0.001
	Cognitive function	0.121	= 0.004
	haPWV	−0.081	= 0.052
hbPWV (*r* = 0.536, adjusted *r*^2^ = 0.279, *P* < 0.001)			
	Body mass index	−0.118	= 0.006
	Strength	0.481	< 0.001
	Flexibility	0.158	< 0.001
	Cognitive function	0.123	= 0.004
	hbPWV	−0.049	= 0.243
baPWV (*r* = 0.541, adjusted *r*^2^ = 0.284, *P* < 0.001)			
	Body mass index	−0.110	= 0.009
	Strength	0.464	< 0.001
	Flexibility	0.157	< 0.001
	Cognitive function	0.121	= 0.004
	baPWV	−0.088	= 0.032

*CAVI, cardio-ankle vascular index; haPWV, heart-ankle pulse wave velocity; hbPWV, heart-brachial pulse wave velocity; baPWV, brachial-ankle pulse wave velocity.*

## Discussion

Significantly higher CAVI and baPWV and a tendency toward higher haPWV were identified in Slow group compared with Fast group and significant decreasing trends were evident in CAVI and baPWV. Nevertheless, significant difference and trend in hbPWV were not found among the tertiles. To our knowledge, this is the first study to directly evaluate the relationship between walking speed and segment-specific arterial stiffness in older adult community dwellers.

During human walking, energy-saving strategy by the pendular mechanism persists (Gomenuka et al., 2014), and the theoretical OWS in elderly is the absolute speed at which participants spend less metabolic energy per meter of traveled distance. In this study, LRI of Slow group shows that observed maximum walking speed may be more limited than OWS (LRI < 100). LRI of Middle and Fast groups was significantly higher than that of Slow group (LRI > 100), and LRI of Fast group notably higher than that of Middle group. Regular exercise intervention can also enhance metabolic economy in elderly (Gomenuka et al., 2020). These results thus suggest that observed maximum walking speed of Slow group is metabolically low economic efficiency to pendular mechanism and may be physiologically limited with aging. In fact, our multiple regression analyses support that BMI, strength, flexibility, and cognitive function affect maximum walking speed.

Blood pressure strongly affects PWV (Benetos et al., 1993; Nishiwaki et al., 2014b, 2017c). In this study, neither systolic BP nor MBP significantly differed among the groups. Here, we assessed arterial stiffness using CAVI, which is theoretically adjusted by BP and represents arterial stiffness from the aorta to the ankle (Shirai et al., 2011). We also adjusted arterial stiffness for BP and other confounding factors using MANCOVA. Thus, these findings indicate that BP in our study did not strongly affect the relationships between walking speed and segment-specific arterial stiffness in older adult community dwellers.

ANOVA and MANCOVA indicate significant differences and trends in baPWV as well as CAVI, but not in hbPWV. Information derived from baPWV is generally considered to be comparable that derived from aortic PWV (Yamashina et al., 2002; Sugawara et al., 2005; Tanaka et al., 2009). However, baPWV moderately reflects arterial stiffness of the lower limbs from the brachium (thoracoabdominal level) to the ankle (Sugawara et al., 2005). Although about 50% of baPWV variances can be explained by central PWV, at least 20% can be explained by leg PWV (Sugawara et al., 2005). Conversely, hbPWV reflects arterial stiffness of the upper limbs from the aorta to the brachium and can serve as a novel marker of arterial stiffness of the proximal aorta (Sugawara et al., 2016, 2019). Our data suggest that the relationship between walking speed and arterial stiffness can vary at different sites, particularly between the upper and central-lower limbs, and walking speed can be mainly associated with arterial stiffness of the central and lower limbs. Indeed, arterial stiffness was inversely related to walking speed in participants with PAD, but not in participants without PAD (Watson et al., 2011). Moreover, single-leg cycling, resistance exercise, and stretching modulate arterial stiffness only in the exercised leg, and not in the control non-exercised leg (Sugawara et al., 2004; Heffernan et al., 2006; Yamato et al., 2017). Therefore, our findings suggest that the relationship between walking speed and arterial stiffness might be contributed to regional relationships or effects rather than systemic relationships or alterations.

We can only speculate on the possibilities underlying the physiological relationship between walking speed and segmental arterial stiffness. Because significantly higher PA and steps were observed in Fast group than in Slow group, repetitive increases in blood flow or shear stress with regular PA may have affected the vascular endothelium or mechanical distension, especially in active parts, which triggers decreased arterial stiffness. From the perspective of functional changes, endothelin-1 and nitric oxide might participate in vascular adaptations to exercise (Tanaka and Safar, 2005; Tanaka, 2019). From the perspective of structural changes, one study also inferred that increased pulse pressure and mechanical distension during aerobic exercise can stretch collagen fibers and modify their cross links, thus reducing arterial stiffness (Tanaka and Safar, 2005; Tanaka, 2019). On the other hand, regular PA or exercise can also maintain or improve walking speed in older adults by improving strength, flexibility, or cognitive function (Miura et al., 2015; Nishiwaki et al., 2018). Indeed, a study shows that an increase in arterial stiffness is associated with low skeletal muscle mass index in community-dwelling older adults, and a close interaction is found between both parameters (Sampaio et al., 2014). Thus, habitual exercise or PA can affect both arterial stiffness and walking speed associated with a maintenance of muscle mass (Gomenuka et al., 2019), indicating a relationship between walking speed and arterial stiffness.

Conversely, age-related increase in arterial stiffness can be greater in aortic and leg than in arm (Avolio et al., 1985; Tomiyama et al., 2003). Upper limb arterial stiffness did not differ significantly among different PA individuals (Aoyagi et al., 2010). That is, even older adults are considered to use upper limb for many times during daily activity (*i.e.*, eating with chopstick, housekeeping, or grooming activities). Although total PA can decrease with aging, the reduction in upper limb activity may be relatively less. Furthermore, walking speed is significantly associated especially with lower strength (Sallinen et al., 2011). These findings suggest that segment of upper limbs than that of central and lower limbs may be less affected by effects of general walking or PA, and thus, hbPWV did not differ significantly among groups. In order to prevent an increase in segment-specific arterial stiffness of the central and lower limbs, our data show the importance of promoting segment-specific exercise training or PA, and thereby maintaining or improving walking speed in older adults.

The studies of Brunner et al. (2011) and Gonzales (2013) examined relationships between gait ability and aortic arterial stiffness in older adults, and indicate that gait ability is related to arterial stiffness for relatively younger older adults with a mean age of sexagenarian. On the other hand, slower gait speed was associated with higher PWV in older adults with PAD, but not in older adults without PAD (Watson et al., 2011). Contrary to these previous studies, in our study, more elderly community dwellers aged 65 to 96 years (76 ± 6 years) were recruited, and thus, many diversities of individuals, such as physical characteristics, lifestyle habit, or physical inactivity, seem likely to be included. Nevertheless, our new finding suggests that maximum walking speed is associated with segment-specific arterial stiffness of the central and lower limbs, but not of upper, in older adult community dwellers with a mean age of septuagenarian.

Walking speed can predict future cardiovascular diseases, disability, or mortality, and is important for frailty prevention in older adults (Fried et al., 2001; Al Snih et al., 2002; Cesari et al., 2005, 2009; Rolland et al., 2006; Ostir et al., 2007; Mozaffarian et al., 2008; Dumurgier et al., 2009; Sattelmair et al., 2010; Shimada et al., 2015). Skeletal muscle strength and body composition are strongly considered as a predictor of impaired walking speed (Misic et al., 2007), which was partially supported by our findings of knee extension strength and BMI. We also found that cognitive function, flexibility, and CAVI or baPWV were independent factors modulating walking speed, but hbPWV was not a significant independent factor. Increased arterial stiffness is widely accepted as an independent risk factor for future cardiovascular diseases or mortality (Laurent and Boutouyrie, 2007; Vlachopoulos et al., 2010, 2012; Ohkuma et al., 2017), and our results of MANCOVA (differences and trends) and multiple regression analyses raise the possibility that a direct relationship between walking speed and arterial stiffness, especially in the central and lower limbs. Thus, the direct relationship between walking speed and arterial stiffness might partially mediate the relationship between gait ability and cardiovascular diseases, disability, or mortality. Such findings from walking speed and arterial stiffness might lead to the development of new strategies to prevent arterial stiffness and gait ability from decreasing or frailty developing in older adults. Our findings may also show the importance of promoting segment-specific exercise training or PA for reducing arterial stiffness and maintaining gait ability.

The strengths of our study include the assessment of walking speed and segmental arterial stiffness in approximately 500 older adult community dwellers, but several important limitations require emphasis. First, our device cannot measure cfPWV at the same time of CAVI, haPWV, baPWV, and hbPWV and need to measure cfPWV after making the switch the measurement mode. Thus, the large population study is difficult to measure or quantify cfPWV by using our device, and we did not directly measure only central and leg PWVs (*i.e.*, carotid-femoral and femoral-ankle). Further detailed studies are needed to examine variations in arterial stiffness at different sites, especially between the upper and lower limbs. Second, the cross-sectional design limits the ability to determine a cause-and-effect relationship regarding the association between walking speed and arterial stiffness. In terms of longitudinal design, further detailed studies are required to elucidate whether walking slowly actually reduces arterial stiffness.

In conclusion, our findings suggest that walking speed is associated with segment-specific arterial stiffness of the central and lower limbs, but not of upper, in older adult community dwellers.

## Data Availability Statement

The raw data supporting the conclusions of this article will be made available by the authors, without undue reservation.

## Ethics Statement

The studies involving human participants were reviewed and approved by the Human Ethics Committee at the Osaka Institute of Technology. The patients/participants provided their written informed consent to participate in this study.

## Author Contributions

MN conceived and designed the study. NO, CN, and MN performed the study. NO and MN analyzed the data and wrote the manuscript. NO, CN, MI, TN, and MN interpreted the data. All authors approved the final version of the article.

## Conflict of Interest

The authors declare that the research was conducted in the absence of any commercial or financial relationships that could be construed as a potential conflict of interest.
